# Special Issue “Cellular and Molecular Insights into Ocular Changes Associated with Systemic Disorders and Conditions”

**DOI:** 10.3390/ijms27042024

**Published:** 2026-02-20

**Authors:** Snježana Kaštelan, Katarzyna Zorena

**Affiliations:** 1 Department of Ophthalmology, Clinical Hospital Dubrava, School of Medicine, University of Zagreb, 10000 Zagreb, Croatia; 2 Department of Immunobiology and Environmental Microbiology, Medical University of Gdańsk, 80-309 Gdańsk, Poland; katarzyna.zorena@gumed.edu.pl

The eye occupies a uniquely strategic and biologically distinct position within the human body. Its combination of transparent optical media, intricate neurosensory architecture, tightly regulated vascular networks, and compartment-specific immunological features creates an organ that is simultaneously exposed to the external environment and closely integrated with systemic physiology. This dual positioning enables ocular tissues to act as high-resolution sentinels for pathological processes across the body. Since the cornea and lens are transparent, the ocular surface is accessible, and the retina and optic nerve are susceptible to systemic insults, the eye offers unique opportunities for in vivo examination of immune, metabolic, neurovascular, and molecular processes that are otherwise difficult to visualise [1,2]. Growing evidence suggests that numerous systemic diseases, including autoimmune disorders, metabolic syndromes, neurodegenerative conditions, genetic syndromes, endocrine abnormalities, cardiovascular diseases, and sleep-related breathing disorders, often exhibit ocular manifestations that may precede extraocular symptoms, highlighting the eye’s potential for early detection and mechanistic insight [1,2,3].

Recent advances in multi-omics profiling, quantitative imaging, computational modelling, and molecular diagnostics have greatly expanded our capacity to leverage ocular tissues as biosensors. Tear proteomics and extracellular vesicle analysis now permit sensitive detection of immune activation and inflammatory shifts, positioning the tear film as a non-invasive “liquid biopsy” for systemic health monitoring [3,4]. High-resolution imaging modalities such as optical coherence tomography (OCT), OCT angiography, adaptive optics, and hyperspectral retinal imaging provide quantitative biomarkers of neurodegeneration, vascular compromise, intracellular defects, and metabolic stress [5]. Cellular and molecular analyses of the corneal epithelium, meibomian glands, retinal microvasculature, crystalline lens, and optic nerve reveal how local alterations reflect systemic pathophysiology. Collectively, these developments are driving a paradigm shift: ocular tissues are not merely passive recipients of systemic disease but active diagnostic and mechanistic interfaces that can guide personalised medical strategies.

The study by Mihaela-Mădălina Timofte-Zorila and colleagues exemplifies the increasing clinical relevance of tear-based diagnostics. By performing a detailed multiplex analysis of Th1/Th2-related cytokines, chemokines, and apoptosis-associated soluble factors in patients with ocular graft-versus-host disease (oGVHD), the authors show that tear biomarkers closely mirror fluctuations in systemic alloimmune activity [6]. Specifically, dynamic modulation of soluble CD27, TRAIL, TRAIL-R2, CCL2, IL-1β, and soluble Fas corresponded with shifts in inflammatory balance and clinical improvement during corticosteroid therapy. These patterns highlight the tear film as a versatile, non-invasive compartment that can capture local and systemic immune transitions with temporal resolution often exceeding standard blood-based assays. The implications extend beyond oGVHD, suggesting tear molecular profiling could serve as an early warning system for autoimmune flares, chronic systemic inflammation, or therapeutic response in disorders such as Sjögren’s syndrome, rheumatoid arthritis, and systemic lupus erythematosus [7,8].

After considering immune signals in tears, the collection turns to genetically precise models that reveal fundamental mechanisms linking intracellular trafficking to systemic phenotypes. Banerjee and colleagues used a vps16-deficient zebrafish model to dissect lysosomal and endolysosomal trafficking defects and their downstream consequences [9]. Loss of Vps16 disrupts intracellular fusion machinery and autophagic flux, leading to systemic abnormalities including hypomyelination, increased neuronal apoptosis, progressive visuomotor decline, and intermediate memory impairment. Mechanistically, defective endolysosomal trafficking leads to accumulation of undegraded substrates, secondary mitochondrial dysfunction, and activation of neuronal death pathways, processes that plausibly explain both central and ocular manifestations. The zebrafish visual system, with its anatomical transparency and conserved development, served as a highly sensitive early indicator of these systemic defects. This study illustrates how genetically tractable models enable the dissection of the molecular and developmental pathways by which systemic genetic disorders manifest in ocular structures, underscoring the value of ocular findings in the preclinical evaluation of gene and molecular therapies for lysosomal storage disorders [10,11].

Complementary mechanistic insight emerges from Keller and collaborators, who characterised thermosensitive transient receptor potential (TRP) channels in human meibomian gland epithelial cells [12]. The study demonstrates functional expression of TRPV1, TRPV3, TRPV4, TRPM8, and TRPV2, showing that channel activation differentially regulates lipid synthesis: stimulation of TRPV1 increases lipid production, whereas activation of TRPM8 decreases it. Mechanistically, TRP channels modulate intracellular Ca^2+^ signalling pathways that control enzymes involved in lipid biosynthesis and secretion, linking temperature sensing and autonomic inputs to glandular output. This framework coherently explains observed relationships between meibomian gland dysfunction and systemic disorders such as diabetes, thyroid disease, and metabolic syndrome [13,14]. Recognising TRP channels as therapeutic targets offers opportunities to precisely modulate lipid secretion in patients whose ocular surface pathology reflects broader systemic dysregulation.

Raju Timsina and colleagues extend the integrative perspective by exploring how variations in membrane cholesterol modulate interactions between αA-, αB-, and αAB-crystallins and model human lens lipid membranes [15]. Their findings challenge the assumption that age-related increases in lens cholesterol are uniformly harmful. Elevated cholesterol concentrations can inhibit excess crystallin binding, thereby preserving membrane permeability to essential antioxidants such as glutathione [16,17]. Given that oxidative stress is a central driver of cataractogenesis and systemic metabolic disorders influence membrane lipid composition across tissues, these results suggest the lens may act as a long-term biochemical archive of metabolic history and a potential site for early metabolic signatures [18].

The Special Issue also includes a comprehensive review by Kaštelan and colleagues that synthesises current understanding of the neuro-ophthalmological sequelae of obstructive sleep apnea (OSA) [19]. OSA, characterised by intermittent upper airway obstruction, recurrent hypoxia, oxidative stress, systemic inflammation, endothelial dysfunction, autonomic imbalance, and intracranial pressure fluctuations, exerts multifaceted effects on the visual system [20,21]. Evidence links these systemic perturbations to compromised retinal ganglion cell health, impaired optic nerve perfusion, and microvascular instability, thereby increasing the risk of glaucoma, optic neuropathy, papilledema, and visual field defects [22,23]. Advanced ocular imaging enables non-invasive detection of subtle structural and microvascular alterations in the retina and optic nerve, creating opportunities for earlier diagnosis and more targeted management of OSA-related morbidity. Given that intermittent hypoxia also contributes to cardiovascular and cerebrovascular disease, the ocular biomarkers discussed have broader systemic implications. This review complements the experimental and mechanistic studies included in the Special Issue by integrating clinical, imaging, and molecular evidence linking intermittent hypoxia and systemic dysregulation to neuro-ophthalmological outcomes. By contextualising ocular findings within broader cardiovascular and neurological pathways, it reinforces the role of ocular biomarkers as integrative indicators of systemic disease burden.

Taken together, the contributions in this Special Issue illustrate the extraordinary potential of ocular biomarkers to deepen our understanding of systemic disease biology. They demonstrate how tear proteomics, genetically tractable models, molecular analyses of glandular physiology, lens biophysics, and advanced imaging can reveal fundamental mechanisms of immune dysregulation, lysosomal impairment, metabolic stress, oxidative imbalance, and neurovascular instability. Importantly, these studies also point toward a future in which ocular assessments become integral to interdisciplinary research frameworks and clinical decision-making.

The next steps for this field require coordinated progress across methodological, computational, and translational domains. One essential direction is to develop integrated multimodal assessment channels that unify tear proteomics, high-resolution imaging, electrophysiology, molecular assays, and functional visual testing. Such unified frameworks could enhance diagnostic accuracy and enable identification of early, convergent disease signatures across tissues. Another important direction is to promote large, longitudinal cohorts designed explicitly to link ocular biomarkers with systemic outcomes. Longitudinal data are vital for distinguishing transient fluctuations from durable disease trajectories and for determining whether ocular signals possess true prognostic value. A further imperative is to leverage artificial intelligence (AI), advanced deep learning approaches, representation learning, and cross-modal fusion models to interpret the vast amount of high-dimensional ocular data now generated in both clinical and experimental settings. AI-enhanced analysis is likely to uncover subtle patterns invisible to human observers, enabling earlier detection and individualised risk stratification.

A final and rapidly expanding direction centres on translational deployment. Advances in microfluidic tear diagnostics, portable multimodal imaging devices, and cloud-based analytic platforms make it increasingly feasible to embed ocular biomarkers into routine clinical workflows, community health programmes, and telemedicine networks. Integrating ocular metrics into systemic disease management has the potential to transform care pathways, including oGVHD monitoring guided by tear biomarkers, metabolic risk screening based on retinal imaging, neurodegenerative surveillance informed by photoreceptor or ganglion cell metrics, and ocular endpoints incorporated into gene therapy trials for lysosomal disorders. By emphasising practical clinical application, researchers can increase visibility of their work, stimulate interdisciplinary collaboration, and enhance the long-term translational impact of ocular science.

In summary, the studies gathered in this Special Issue demonstrate that the eye is not merely a sensory organ but a powerful biological interface capable of revealing dynamic systemic processes. Its accessibility, neurosensory sensitivity, and molecular diversity position it as a leading candidate for next-generation diagnostic and mechanistic tools. As methodologies mature and cross-disciplinary collaborations deepen, ocular biomarkers are poised to become a foundational component of precision medicine, bridging ophthalmology with immunology, neurology, endocrinology, genetics, and cardiovascular science. By embracing integrated analytics, translational deployment, and longitudinal validation, the field can unlock the full potential of ocular tissues as comprehensive sensors of systemic health. We hope that this Special Issue will stimulate interdisciplinary collaboration and encourage researchers to integrate ocular biomarkers into systemic disease research and clinical decision-making, thereby supporting the continued convergence of ophthalmology, molecular medicine, and precision health within an integrated systems-based framework.
